# Terabase-scale metagenome coassembly with *MetaHipMer*

**DOI:** 10.1038/s41598-020-67416-5

**Published:** 2020-07-01

**Authors:** Steven Hofmeyr, Rob Egan, Evangelos Georganas, Alex C. Copeland, Robert Riley, Alicia Clum, Emiley Eloe-Fadrosh, Simon Roux, Eugene Goltsman, Aydın Buluç, Daniel Rokhsar, Leonid Oliker, Katherine Yelick

**Affiliations:** 1 Computational Research Division, Lawrence Berkeley National Laboratory, Berkeley, CA 94720 USA; 2 Joint Genome Institute, Lawrence Berkeley National Laboratory, Berkeley, CA 94720 USA; 3 Parallel Computing Lab, Intel Corp., Santa Clara, CA 95054 USA; 4 Department of Electrical Engineering and Computer Sciences, University of California, Berkeley, CA 94720 USA; 5 Department of Molecular and Cellular Biology, University of California, Berkeley, CA 94720 USA

**Keywords:** Genome assembly algorithms, Software, Metagenomics

## Abstract

Metagenome sequence datasets can contain terabytes of reads, too many to be *coassembled* together on a single shared-memory computer; consequently, they have only been assembled sample by sample (*multiassembly*) and combining the results is challenging. We can now perform coassembly of the largest datasets using *MetaHipMer*, a metagenome assembler designed to run on supercomputers and large clusters of compute nodes. We have reported on the implementation of *MetaHipMer* previously; in this paper we focus on analyzing the impact of very large coassembly. In particular, we show that coassembly recovers a larger genome fraction than multiassembly and enables the discovery of more complete genomes, with lower error rates, whereas multiassembly recovers more dominant strain variation. Being able to coassemble a large dataset does not preclude one from multiassembly; rather, having a fast, scalable metagenome assembler enables a user to more easily perform coassembly and multiassembly, and assemble both abundant, high strain variation genomes, and low-abundance, rare genomes. We present several assemblies of terabyte datasets that could never be coassembled before, demonstrating *MetaHipMer*’s scaling power. *MetaHipMer* is available for public use under an open source license and all datasets used in the paper are available for public download.

## Introduction

A metagenome is a representation of the genomic content of a soil, water or other environmental sample. Metagenome assembly is challenging due to sequencing error, repetitive content, and library and sequencing bias. In addition, a metagenome sample can contain many thousands of different genomes with varying degrees of similarity, sometimes sharing genetic material, and occurring at vastly different abundances. Advances in sequencing technology make it possible to sample low abundance organisms but pose additional challenges for assembly, with datasets on the order of terabytes, too big to be assembled as one set of reads by assemblers that only run on shared memory machines. This has led to various approaches which either reduce input size by filtering^1^ or combine results of multiple partial assemblies^2,3^. Many authors have attempted to estimate sequencing effort required to recover the majority of organisms in complex communities^4–7^. However, limitations in assembler design have prevented testing these models since it has been possible for some time to produce more sequence data than can be assembled in a single shared memory computer.

In this paper we show that assembling all the reads from a project or sample together (*coassembly*) has benefits that cannot be realized by combining the results of multiple partial assemblies (*multiassembly*). To achieve coassembly for terabase-scale metagenomic datasets we have developed *MetaHipMer*, a metagenome assembler able to run on high-performance supercomputers. *MetaHipMer* can run effectively on a single node or a small cluster, but importantly, can also scale to thousands of compute nodes, allowing it to utilize potentially petabytes of memory to assemble terabase-scale or larger metagenomes. *MetaHipMer* assemblies can be completed rapidly, on the order of minutes or hours for multiple terabytes of data. The technical details of the parallelization approach for *MetaHipMer* have been described in detail elsewhere^8^; here we focus on the benefits of coassembly of terabase-scale datasets.

We show several advantages of coassembly over multiassembly for a large marine dataset. Coassembly recovers a larger fraction of the input genomes than multiassembly, especially for low abundance species. Furthermore, the sequence duplication present in a multiassembly leads to error rates up to 3.5x higher and state-of-the-art deduplication tools do not resolve that problem. We also investigate a disadvantage of coassembly, namely poor recovery of strain variation^9^. As expected, coassembly recovers smaller fractions of strains than multiassembly. However, with a fast, scalable assembler, terabase-scale datasets can be both coassembled and rapidly multiassembled to give complete assemblies of both dominant strain variation and rare species.

## Results

We investigate coassembly and multiassembly using a large (822 GB) marine dataset, both with and without the injection of synthetic reads, and analyze various aspects of coassembly versus multiassembly, including MAG discovery, assembly quality, and the impact of strain variation. In addition, we compare *MetaHipMer* against two of the most commonly used assemblers, MEGAHIT^10^ and metaSPAdes^11^, and show that *MetaHipMer* produces equivalent quality assemblies on a variety of different datasets. Finally, we describe several new large-scale metagenomes recently assembled with *MetaHipMer* that could not be done before, ranging from 2.6 TB of sequencing data for a time series of wetlands soil samples to 3.3 TB for soil samples in carbon cycle experiments. These assemblies are too big to be coassembled on a shared-memory computer, but they could all be assembled in a matter of hours using NERSC’s Cori supercomputer^12^.

### Comparing coassembly to multiassembly

To explore the benefits of coassembly of very large scale datasets, we used the *WA* dataset, which is a collection of marine microbial communities from the Western Arctic Ocean, and consists of 822 GB of 2.5 billion reads in 12 samples, with a read length of 150 and average insert size distribution of 270±30. Additional details of the WA dataset and all others used in this paper can be found in the Supplementary information. We ran *MetaHipMer* (v1.2.1) on the Cori supercomputer^12^, performing both a coassembly of all 12 samples together, and an asssembly of each sample individually, and we combined the individual sample assemblies to form a multiassembly.

**Figure 1:**
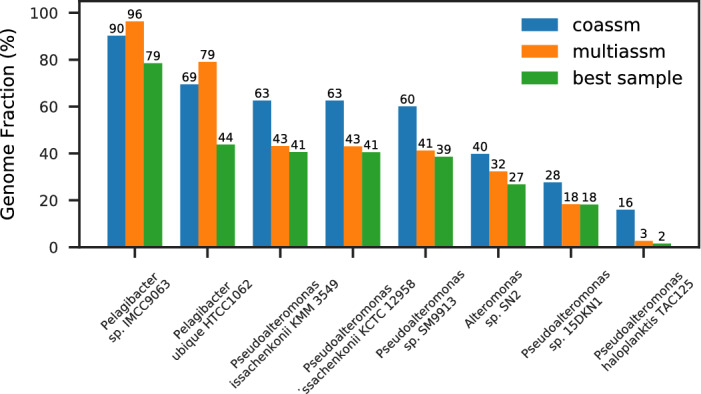
Genome fractions for references from MarRef found in the WA assemblies.

**Figure 2:**
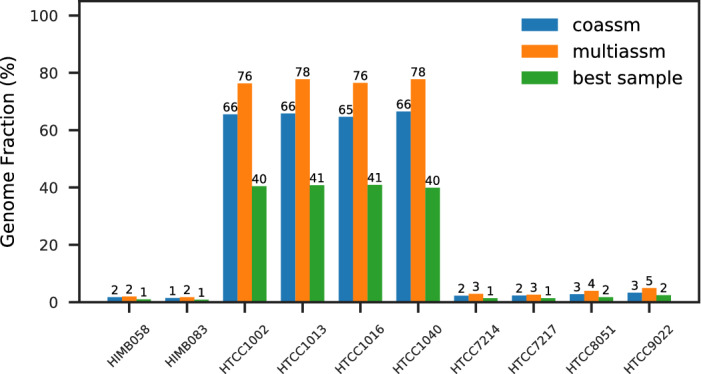
Genome fractions for strains of *P. ubique* found in the WA assemblies.

We used MetaQUAST^13^ to evaluate the assemblies, using as references all 971 genomes in the MarRef marine genomes database^14^. The parameters used for MetaQUAST and all other software tools can be found in the Supplementary information. We found traces of 871 genomes in the coassembly, with 40 of those exceeding a 1% genome fraction, and 8 with at least a 10% genome fraction; these are shown in Fig. 1. The series marked “best sample” are the highest genome fractions for any single sample assembly. Most of the genomes recovered at above 10% are of the *Pseudoalteromonas* genus, with two strains of the same species (*P. issachenkonii*). For all of these, coassembly typically recovers 40–50% more than multiassembly. However, for the two largest fraction genomes recovered (of the *Pelagibacter* genus), multiassembly recovered a slightly higher fraction (up to 14% more) than coassembly, which is likely a consequence of the strain variation in these dominant genomes. The MarRef database has only one strain of *P. ubique*, so to further investigate the issue of strain variation, we used an additional 10 strains of *P. ubique* as references for another MetaQUAST assessment. It can be seen in Fig. 2 that multiassembly recovers a higher genome fraction for all strains than coassembly (up to 18% more), but coassembly still manages to recover the same set of strains as multiassembly.

To assess the presence of unlabeled MAGs (not from known references), we ran MetaBAT2^15^ on the assemblies and used CheckM^16^ to assess the quality of the bins discovered. We used the MetaBAT R tool^17^ to determine the number of medium quality MAGs (completion $\ge$50% and contamination $\le$10%)^18^. When contamination due to strain heterogeneity is filtered out (the strain removal flag is set to true), the number of MAGs is almost identical between coassembly (31) and multiassembly (30). Without strain heterogeneity filtering, there are 26 medium quality MAGs in the coassembly, and only 1 in the multiassembly. This is to be expected: in the coassembly, out of 77 bins with completion $\ge$50% there are 21 with a strain heterogeneity $\ge$50%, whereas in the multiassembly, out of 109 bins with $\ge$50% completion, there are 75 with strain heterogeneity $\ge$50%. Thus it is clear that coassembly removes strain information.

To explore the impact of coassembly on low abundance genomes, we injected a set of synthetic reads into the WA dataset, following an approach similar to Wang et al.^19^. The genomes for generating the synthetic reads were drawn from the MarRef database, and were selected based on two criteria: first, they are Arctic ocean genomes, and second, they were found in the WA dataset with the MetaQUAST analysis, but at low genome fractions (generally under 1%). Thus we have a set of genomes (25 were found) that are realistic constituents of the WA community, but are of low enough abundance that we can better analyze the effects of sequencing depth on the various assembly approaches. We used CAMISIM^20^ to generate a set of 12 synthetic metagenome samples (one per sample of WA), with the *replicate* model and the default lognormal abundance distribution. This is similar to the way the first CAMI challenge’s high complexity dataset was generated^21^. Each generated sample was injected into one of the WA samples to create the *WAmix* dataset.

Running *MetaHipMer* on the Cori supercomputer^12^, we assembled the complete dataset of all 12 samples of WAmix together (coassembly), and we assembled each individual sample separately. We then assessed the quality of the assembly in terms of the known 25 references, using MetaQUAST. Table 1 shows results for several different assemblies: the *coassembly*; the *multiassembly*, with all 12 samples combined; a *deduplicated* version of the multiassembly (using bbtools dedupe^22^); the average across all single samples (*single sample avg*); and the synthetic reads only, i.e. not injected into WA (*ArcticSynth*). As expected, the duplication ratio is very high for the multiassembly (7.3), and although deduplication helps, it is clearly difficult to remove duplicates, because the ratio is still high, at 4.3. Note that the duplication ratio is the total number of bases in the assembly aligned to the reference, divided by the number of bases in the reference. A consequence of these highly duplicated assemblies is that misassemblies are also high; as expected, the number of extensive misasssemblies in multiassembly (365) is roughly 12x that of the single sample average (30). By contrast, the coassembly has a far lower number of extensive misassemblies (96), which is only double that of the purely synthetic assembly (45), and the duplication ratio (1.2) is much lower than the deduplicated multiassembly. Also, the coassembly captures the same fraction of the genomes as the purely synthetic assembly (94%), whereas the multiassembly is missing a large fraction of the genomes, capturing around 46%.

**Table 1:** Assembly quality for WAmix.

Sample type	Length (gbp)	Largest alignment	Contigs (millions)	Genome %	Misassemblies		Mismatches/100 kbp	Indels /100 kbp	Duplication ratio
					Extensive	Local			
Coassembly	7.4	1,085,233	5.5	94	96	58	159	3.3	1.2
Multiassembly	12.0	50,429	10.2	46	365	396	1654	66.1	7.3
Deduplicated	8.6	50,429	7.0	45	243	294	1146	59.6	4.3
Single sample avg	1.0	29,207	0.8	24	30	33	264	10.6	1.2
ArcticSynth	0.1	1,085,223	0.01	94	45	15	26	1.5	1.0

**Figure 3:**
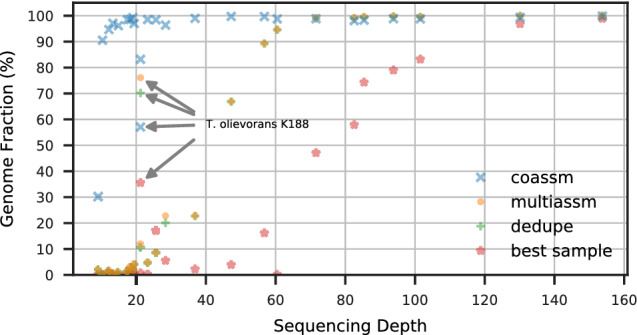
Genome fraction vs depth for synthetic reference genomes within WAmix.

The coassembly captures much higher genome fractions of low depth genomes than multiassembly, as can be seen in Fig. 3, which shows the fractions of the assembly that align to the various depths of reference genomes. For low depths (under 20), the coassembly usually captures most of the genome (above 90%), whereas the multiassembly captures very little of the genome (under 5%), and it only does as well as the coassembly when the depth reaches 70. The multiassembly is still an improvement over one sample, which only exceeds 90% at depths greater than 130. There is one notable exception (labeled on the figure): for *T. oleivorans* strain K188 at depth 21, coassembly recovers 57% whereas multiassembly recovers 76%. There is another, more abundant strain of *T. oleivorans* (strain R6 15) at a higher depth (85), and it is likely that the overlap between these two strains is what makes coassembly perform worse, since it is unable to capture both strains fully.

**Figure 4:**
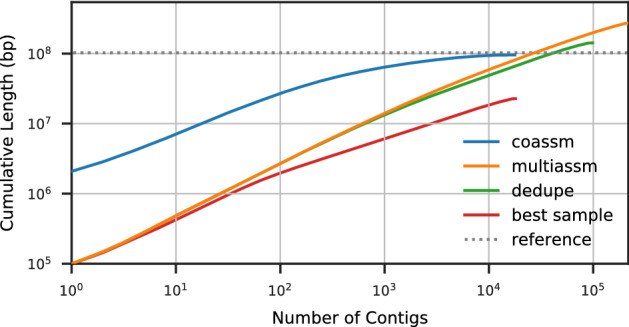
Cumulative lengths for contigs aligned to synthetic reference genomes within WAmix.

The coassembly also improves contiguity, as can be seen in Fig. 4, which shows a cumulative length plot of all sequences that align to the synthetic reference genomes, broken at misassemblies. The cumulative length is computed with the contigs ordered from longest to shortest. The multiassembly is much more fragmented than the coassembly, and has contiguity not much better than a single sample. Because the contigs are sorted by length, we can see that the longest contigs (on the left hand side of the x-axis) produced by coassembly are at least an order of magnitude longer than the ones produced by multiassembly. Although the multiassembly gives an overall larger cumulative length (the right hand side of the x-axis in Fig. 4), these results do not reflect useful information because they are due to the duplications (everything above the reference dotted line represents duplications).

Related to the issue of strain diversity is *chimericity*^23^, where a contig comprises reads from multiple genomes. The impact of coassembly on chimericity is unknown, so we calculated the chimericity for the synthetic reads in the WAmix dataset. Chimericity is computed as entropy, i.e. $chimericity=\sum _i p_i log(p_i)$, where $p_i$ is the proportion of reads that came from genome *i*^24^. Multiassembly is slightly worse for chimericity, with an average over all contigs of 0.011, compared to 0.005 for coassembly. However, this result is a consequence of the fact that there are more chimeric contigs in multiassembly (2.2%) compared to coassembly (1%); the average chimericity over the chimeric contigs is 0.49 for multiassembly and 0.52 for coassembly. We conclude that coassembly does not substantially increase chimericity, and chimeric contigs remain rare.

### Quality comparisons

There have been many assembler comparison studies (e.g. ^19,25,26^). These have demonstrated that different assemblers have different strengths and weaknesses. Here we compare *MetaHipMer* to two of the most commonly used assemblers: MEGAHIT^10^ and metaSPAdes^11^. The comparison here does not purport to be extensive; rather it is intended to demonstrate that *MetaHipMer* produces assemblies that are roughly equivalent in quality to those produced by the other assemblers on datasets small enough to assemble on a single shared memory computer.

All the assemblers were run in their default settings and no attempt was made to tune them for the given datasets. In practice, it is often the case that an assembler can be tuned for a given dataset, which may make one assembler work better than others for particular datasets. However, for assemblers that take a long time to run, there is often not the luxury of repeated runs to fine-tune the parameters. For clean comparisons, we do not do any pre- or post-processing of the data. Of course, in practice, many assembly pipelines use extensive pre- and post-processing, which can improve assembly quality or reduce running time.

#### Datasets

The quality comparisons involve the assemblies of four different datasets, two that are purely synthetic (ArcticSynth and MBARC-26), and two that are mixed real world and synthetic (Gut and Marine):

- *ArcticSynth*. This consists of only the synthetic references from WAmix. As noted before, 25 references were selected from MarRef, and we used CAMISIM to generate a total of 32 million synthetic reads (9.9 GB) of length 150 bp, in 12 replicate samples with a lognormal abundance profile. All samples were combined into a single dataset. The average insert size was 270 and the standard deviation was 30.
- *MBARC-26*. This is a synthetic high-depth, simple dataset composed of 23 bacterial and 3 archaeal strains with finished genomes that span 10 phyla and 14 classes, a range of GC contents, genome sizes, repeat content, and that encompass a diverse abundance profile^27^. The dataset comprises 173 million (32 GB) 150 bp paired-end reads with average insert size of 270 and standard deviation of 30.
- *Gut*: This is a mixed dataset, with 101 bp-length paired-end reads, drawn from real sequencing of the human gut microbiome, and injected synthetic reads from 5 other genomes^19^. Injection of all the genomes was done at depths 5x, 20x and 50x. Each one was evaluated separately. The dataset sizes, respectively, are 31 million reads (6.8 GB), 33 million reads (7.3 GB), and 37 million reads (8.2 GB). The real gut dataset without injections is 30 million reads (6.7 GB).
- *Marine*: This is another mixed dataset, with 101 bp-length paired-end reads drawn from the sequencing of a Tara Oceans Polar Circle DNA sample, and injected synthetic reads from 8 other genomes^19^. Injection of all the genomes was done at depths 5x, 20x and 50x. Each one was evaluated separately. The dataset sizes, respectively, are 480 million reads (101 GB), 483 million reads (102 GB), and 489 million reads (104 GB). The real marine dataset without injections is 479 million reads (101 GB).

#### Assembly quality evaluation

Table 2 shows the quality results for the assemblies of the synthetic datasets (ArcticSynth and MBARC-26). We assessed the completed assemblies using MetaQUAST and focus on several different aspects: *contiguity*, as measured by the NGA50 (computed over the combined references) and the largest alignment; *genome fraction* recovered, as measured by the percentage of the reference aligning to the assembly; *errors*, as measured by misassemblies, mismatches and indels; and revealed *diversity*, as measured by genome bins and rRNA counts. For the rRNAs, we focus on the 16S and 23S (since the 5S are very short ${-}$ 120bp), and only report the complete rRNAs, as determined by barrnap^28^. The metaSPAdes assembly has noticeably lower rRNA discovery than the other two. This is a trend consistent across all datasets. The genome bins were determined using the same process with MetaBAT/CheckM as described before; reported here are the medium quality counts, where medium quality is defined according to the MAG standard as completion $\ge$ 50% and contamination $<$ 10%. These counts are similar except for metaSPAdes, which has much lower counts (4 and 8) compared to the other assemblers (from 15 to 19).

**Table 2:** Quality of assemblies of synthetic datasets.

Assembler	NGA50 (kbp)	Largest alignment	Contigs	Genome %	Misassemblies		Mismatches/100 kbp	Indels /100 kbp	Genome bins	rRNAs	
					Extensive	Local				16S	23S
*ArcticSynth*											
MetaHipMer	52	1,085,233	9060	93.8	45	15	26	1.5	15	13	11
MEGAHIT	65	1,085,427	7432	95.1	110	78	134	1.9	16	14	7
MetaSPAdes	17	1,290,245	17,701	91.2	53	50	291	1.9	8	7	3
*MBARC-26*											
MetaHipMer	152	2,055,376	4239	92.3	80	30	3	0.4	19	15	11
MEGAHIT	121	1,636,294	5371	93.1	69	34	8	0.7	19	16	12
MetaSPAdes	193	2,055,367	4987	92.1	76	58	69	3.3	4	6	4

**Figure 5:**
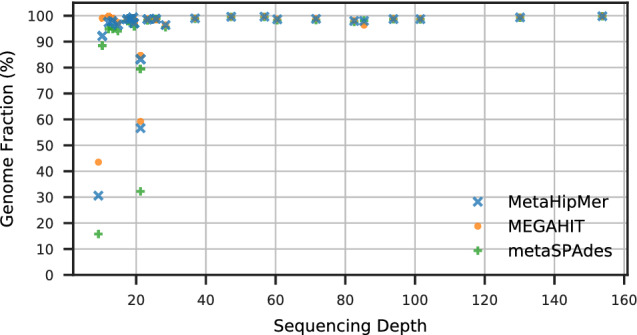
Genome fraction vs depth for assemblies of the ArcticSynth dataset.

**Figure 6:**
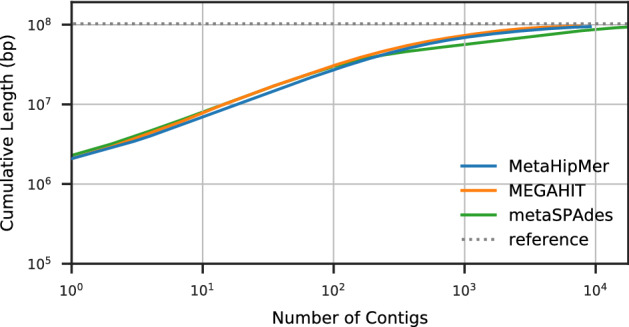
Cumulative lengths for contigs for assemblies of the ArcticSynth dataset.

The comparisons between the assemblers on ArcticSynth are further illustrated in Figs. 5 and 6. The impact of depth on the genome fraction is shown in Fig. 5; generally genome fraction is close to 100% for higher depths and only low when considering very low depth or non-dominant strains. The genome fraction recovered is similar across the different assemblies. Figure 6 shows the cumulative lengths of the contigs that align to the references for ArcticSynth, broken at misassemblies, and ordered from the longest to the shortest. The extent of the x-axis data indicates the total number of contigs, and the extent of the y-axis data indicates the total assembled length, which is related to the genome fraction and the duplication ratio. The three assemblers are generally equivalent in contiguity, which is dominated by the assembly of high abundance genomes.

The quality assessments for the assemblies of the mixed synthetic/real results are shown Table 3 for Gut and in Table 4 for Marine. In general, the MEGAHIT assemblies tend to have more misassemblies, resulting in lower NGA50s (similar to the trend seen for the assemblies of the synthetic datasets), and once again, the metaSPAdes assemblies have the lowest rRNA discovery (and in the case of Gut, the lowest count of genome bins). At low depth (5x), the metaSPAdes assemblies have the best genome fraction, whereas the *MetaHipMer* assemblies are more conservative, and consequently have reduced genome fraction, but fewer misassemblies.

**Table 3:** Quality of Gut mixed assemblies for various depths.

Assembler	NGA50 (kbp)	Largest alignment	Contigs	Genome %	Misassemblies		Mismatches/100 kbp	Indels /100 kbp	Genome bins	rRNAs	
					Extensive	Local				16S	23S
*5x*											
MetaHipMer	0.13	12,213	82,299	81.8	23	3	77	10.0	8	18	8
MEGAHIT	0.14	37,232	94,473	82.8	48	15	66	4.1	12	5	6
MetaSPAdes	0.15	32,154	106,047	87.4	29	9	94	6.3	10	5	6
*20x*											
MetaHipMer	127	667,522	74,209	99.1	8	3	17	0.9	14	15	11
MEGAHIT	88	391,021	86,687	98.3	30	10	22	1.6	17	3	7
MetaSPAdes	138	458,963	97,852	99.1	1	6	13	1.8	9	3	6
*50x*											
MetaHipMer	150	667,521	73,881	98.6	5	4	14	0.6	13	19	12
MEGAHIT	111	666,969	86,608	98.7	18	7	21	1.8	16	5	8
MetaSPAdes	150	1,365,043	97,869	99.2	0	6	14	1.8	9	2	6

**Table 4:** Quality of Marine mixed assemblies for various depths.

Assembler	NGA50 (kbp)	Largest alignment	Contigs	Genome %	Misassemblies		Mismatches /100 kbp	Indels/100 kbp	Genome bins	rRNAs	
					Extensive	Local				16S	23S
*5x*											
MetaHipMer	0.18	28,718	196,390	88.2	3	95	72	2.4	7	12	6
MEGAHIT	0.24	34,471	197,872	90.5	68	27	71	2.5	9	15	8
MetaSPAdes	0.36	28,334	158,348	95.6	23	24	96	4.2	12	1	6
*20x*											
MetaHipMer	251	1,238,326	179,308	99.6	4	10	16	0.2	11	20	14
MEGAHIT	239	1,382,528	184,773	99.7	3	15	24	1.3	13	15	9
MetaSPAdes	249	1,382,094	147,628	99.5	0	20	12	0.7	14	1	7
*50x*											
MetaHipMer	328	1,410,119	181,509	99.6	6	7	15	1.3	12	21	12
MEGAHIT	239	1,410,120	184,646	99.7	6	16	24	1.2	12	17	9
MetaSPAdes	246	1,382,118	147,619	99.8	0	24	16	1.2	12	1	6

#### Assembler running times

*MetaHipMer* is designed to run on distributed memory high performance supercomputers, such as NERSC’s Cori system. This enables us to complete very large assemblies, such as those described in a previous section. By contrast, the other assemblers we have compared against can only run on single server shared memory systems. Although *MetaHipMer* is designed for supercomputers, it still runs efficiently on a single shared memory computer, and can be used on a variety of hardware platforms, and has the potential to be used on cloud systems, such as Microsoft Azure^29^. For comparison of running times and resource utilization, *MetaHipMer* was thus also run on a single server shared memory system.

Table 5 presents results of running the three assemblers on a single server with four 20-core (total 80) Intel Xeon E7-8870 2.10 GHz processors, and 1 TB of RAM, for the test datasets. We can see that although *MetaHipMer* is designed to run on distributed memory systems, its performance on a single shared-node machine is still reasonable, being about one-third to two times slower than MEGAHIT and about two to five times faster than metaSPAdes. Because we have ample memory on the distributed systems, *MetaHipMer* has not been optimized extensively for memory use; hence the memory usage is higher than the other assemblers.

**Table 5:** Running time (minutes) and memory usage (GB) of the assemblers.

Assembler	ArcticSynth		SYNTH64D		MBARC-26		Gut 50x		Marine 50x	
	Time	Memory	Time	Memory	Time	Memory	Time	Memory	Time	Memory
MetaHipMer	20	93	24	100	153	281	22	119	203	614
MEGAHIT	22	4	15	4	90	42	16	4	143	42
MetaSPAdes	101	76	101	76	347	129	80	42	403	128

*MetaHipMer* can utilize petabytes of distributed memory to assemble very large datasets, and it does so with efficient use of compute resources. To demonstrate this, we assembled the Marine 50x dataset on a cluster comprised of Xeon Phi processors (the Knights Landing–KNL–partition of the NERSC Cori system). The assembly takes 100 min at 8 nodes (the minimum required because of memory constraints—each node has 96 GB RAM), 31 min at 32 nodes and 20 min at 64 nodes. This is a scaling efficiency of 80% from 8 to 32 nodes, and 64% from 8 to 64 nodes.

In summary, we have shown that the quality of assemblies produced by *MetaHipMer* is competitive with MEGAHIT and metaSPAdes assemblies. And although *MetaHipMer* can be run on a single server with comparable running times to other assemblers, it has the unique capability to scale efficiently on a distributed memory supercomputer.

### New terabase-scale assemblies

*MetaHipMer* has already enabled the coassembly of several large scale datasets that could not be assembled before. A few of these are described here; see Table 6 for some of their characteristics. In the original publication for the *cow rumen* dataset, the sequences were assembled individually, not as a single coassembly. The running times for all the assemblies were on 1024 nodes on the Cori supercomputer, KNL partition. Note that although the *cow rumen* dataset includes long reads, we did not use these because *MetaHipMer* is a short-read assembler.

**Table 6:** New terabase-scale dataset assemblies. Columns labeled * are calculated for scaffolds $\ge 500bp$.

Description	Data	Assembly$^{*}$	Scaffolds$^{*}$	N50$^{*}$	Time
	(TB)	(gbp)	(millions)	(kpb)	(hrs)
*Wetlands*: Metagenomics sequences from a time-series of wetlands soil samples collected from several physical sites in the Twitchell Wetlands in the San Francisco Bay-Delta^30^	2.63	46.2	41.6	1.2	5.14
*Cow Rumen*: A collection of metagenomic DNA sequenced from microbes adherent to plant fiber incubated in the cow rumen^31^	2.66	18.4	12.7	1.7	2.10
*Soil Carbon*: Metagenome DNA sequenced for a project that aims to identify and characterize the dominant uncultivated microorganisms that mediate major transformations in the soil carbon cycle^32^	3.34	15.1	15.5	1.0	3.20

## Discussion

For the analysis of the impact of coassembly, we compared the coassembly to a simple case of performing multiple individual assemblies of each sample and then concatenating the results. We attempted to improve the multiassembly by running *dedupe* to automatically remove duplicated sequences, but found that although the duplication ratio was reduced from 7.3 to 4.3, it was still very high and the misassemblies remained high. Clearly, there could be better ways of producing the multiassembly than simple concatenation, but exploring this is still an open research topic^9^. With the ability to do complete coassemblies of all the data, there is less of a need to improve the quality of multiassemblies.

For our comparisons, we made no attempt to tune the different assemblers, and simply used the default parameter settings. It is highly likely that careful tuning for particular datasets will improve the assembly qualities; however, tuning is expensive and can require multiple runs, which can be prohibitive with an assembler than runs on a single shared-memory computer and takes hours to complete. This points to another benefit of fast assemblies on distributed memory systems: if an assembly takes on the order of minutes instead of hours, it is reasonable to do multiple runs to explore the parameter space and find the best settings possible. With *MetaHipMer*, the ability to do runs very quickly makes it possible to do parameter sweeps, and potentially tune the assembly in different ways on different runs for different outcomes, for example, one run for contiguity, one for minimal errors, etc.

Based on the data presented here and our experience with combined assembly of metagenomes, we are not suggesting that metagenomes should *only* be coassembled. There are cases when multiassembly would be preferable, for example, to recover strain variation in high abundance genomes. Of course, being able to coassemble a large dataset does not preclude one from multiassembly; rather, having a fast, scalable metagenome assembler enables a user to more easily perform both coassembly and multiassembly, and assemble both abundant, high strain variation genomes, and low-abundance, rare genomes.

## Conclusions

We have explored the trade-offs between coassembly and multiassembly using *MetaHipMer*, a new distributed memory metagenome assembler that can scale to thousands of compute nodes in a supercomputer or compute cluster. Our results showed that on a mixed dataset combining both real-world and synthetic reads, coassembly recovers a larger fraction of both synthetically injected genomes and real genomes than multiassembly, especially for low-depth genomes. The only exception is for high abundance strains, where multiassembly recovers more strain information. Furthermore, the duplication present in a multiassembly leads to much higher error rates (up to 3.5x higher) and state-of-the-art deduplication tools do not significantly address that problem. We also compared *MetaHipMer* to two leading assemblers, MEGAHIT and metaSPAdes, on smaller assemblies. We analyzed the quality in multiple dimensions, including contiguity, accuracy (errors), feature recovery and genome fraction, and found that *MetaHipMer* produces assemblies of similar quality to the other two assemblers. Finally, we described the results of a number of new assemblies performed by *MetaHipMer* that are too large to be coassembled on a single shared-memory computer; these results demonstrate the potential for new scientific discovery enabled by the massive computational power harnessed by *MetaHipMer*.

## Methods

Most of the implementation of *MetaHipMer* has been described in detail previously^8,33^. We recapitulate this description at a high level here for clarity. There are two new parts, however, compared to our previous presentations. First, *k*-mer analysis was previously implemented in MPI using collectives; here we describe a new implementation in UPC++. Second, we present an entirely new scaffolding module, called *cgraph*, which was developed specifically to target metagenomes, and improves both the quality of the assemblies and the parallel scalability relative to the previous scaffolder.

*MetaHipMer* can be split into two broad phases: first, contigs are iteratively constructed using de Brujin graphs (similar to the way the IDBA-UD^34^ and MEGAHIT^10^ assemblers work), and, second, in scaffolding, the contigs are joined together to resolve repetitive genomic regions and further increase contiguity. Both iterative contig generation and scaffolding employ distributed memory parallelization via UPC^35^ and UPC++^36^. Unlike popular send/receive message passing systems, UPC and UPC++ allow a thread on one processor to directly read or write the memory or execute a function on another processor, even when the two processors are on different network-connected nodes.

### Iterative contig generation

**Figure 7:**
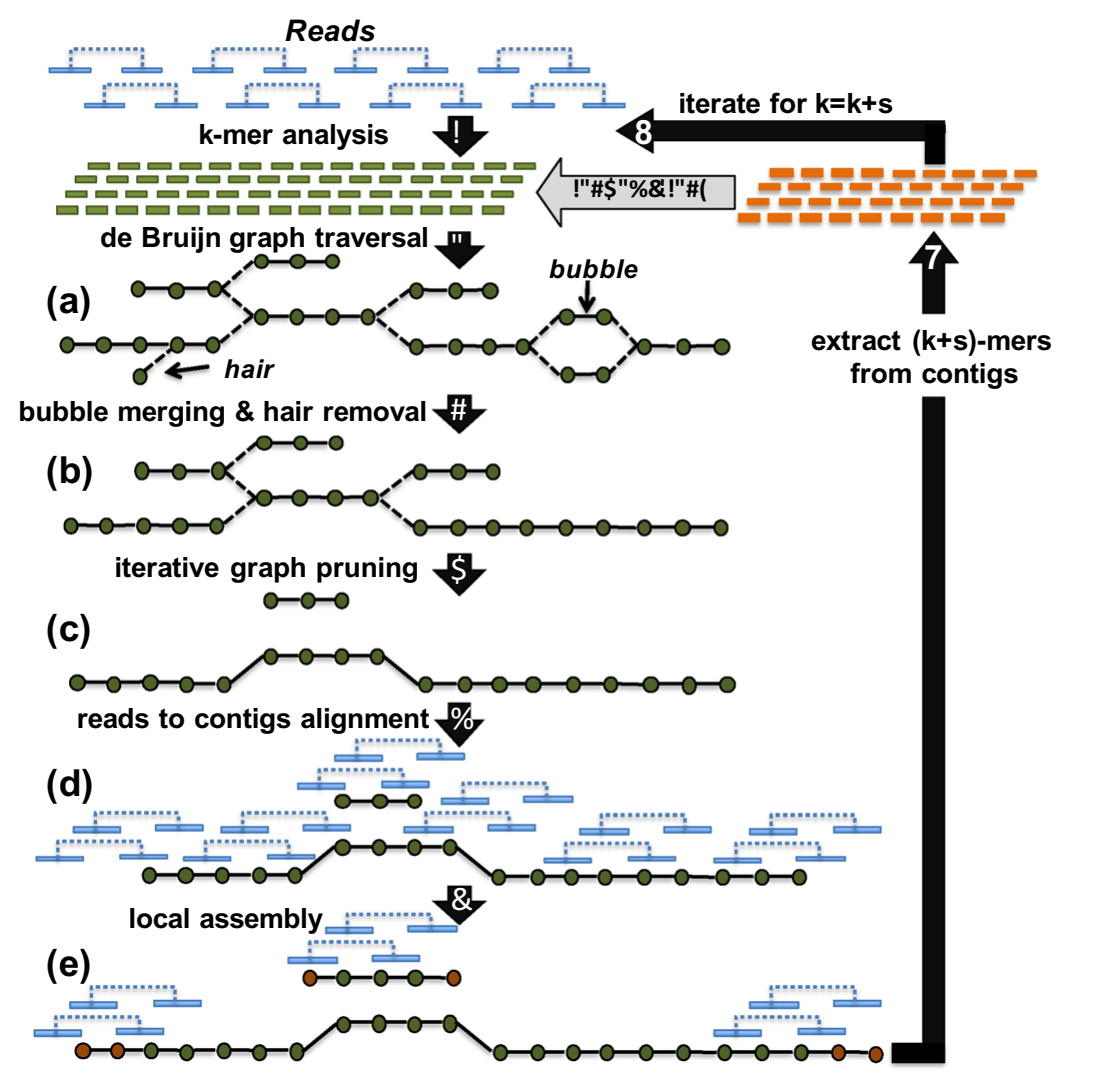
Iterative contig generation workflow in *MetaHipMer*. Image source: Georganas et al.^8^. Reproduced under a CC BY 4.0 open access license by permission of E. Georganas.

The de Bruijn graph approach relies on *k*-mers, which are subsequences of length *k*, extracted from reads. In general, a higher value of *k* is better because it produces longer *k*-mers that can span repeats. However, a metagenome dataset will likely contain some species that have been sampled at low abundance, and a large *k* will lead to a very fragmented graph and low contiguity. Thus there is a trade-off in *k*-mer size that affects high and low frequency species differently. The solution employed by *MetaHipMer* and other assemblers is to iterate through repeated contig constructions, with increasing values of *k*^10,11,34^. Each iteration (for a given value of *k*), produces a set of contigs that is then used in the next iteration to add *k*-mers (the contigs are treated as error-free reads). This iterative process is shown in Fig. 7 (reproduced from Georganas et al.^8^). In this way, low abundance species are assembled in the early iterations, and more repeats are resolved in the later iterations.

There are multiple steps within each contig generation iteration. First, in *k*-mer analysis (step 1 in Fig. 7), overlapping *k*-mers are extracted from reads, and only those occurring more than once are retained to filter out sequencing errors. In addition, all possible extensions (single nucleotides) on either side of a *k*-mer are recorded, provided they are *high quality*, i.e. they occur at least twice and are of sufficient sequencing quality (as reported by the read quality scores). In *MetaHipMer*, we use a parallel implementation of *k*-mer analysis in UPC++ (this is a new implementation, not previously described). Each rank processes a subset of the reads and splits them into *k*-mers, which are then stored, via *remote procedure call* (RPC) updates, in a global hash table. Each rank can then access its local portion of the global hash table to count the *k*-mers independently, and discard those that are infrequent and hence likely errors. Because this approach filters out errors *MetaHipMer* does not require preprocessing to remove errors from reads (which is often a computationally expensive operation). Similar to the MPI implementation described in HipMer^37,38^, the *k*-mer analysis also uses distributed Bloom filters to reduce the memory explosion that is induced by erroneous *k*-mers.

In the second step (2 in Fig. 7), contigs are formed by traversing the de Bruijn graph of *k*-mers, which is represented as a distributed hash table. During a traversal, *MetaHipMer* uses the count of an extension to determine whether to extend the contig or abort the traversal. If an extension is the only one with a count that exceeds a threshold, $t_{hq}$, then it is chosen. If no extension count exceeds the threshold, there is a deadend in the graph, whereas if multiple extension counts exceed the threshold then there is a fork (the dashed lines in (a) in Fig. 7). Because metagenomes consist of many different sampling abundances, the threshold is adaptive, and depends on the frequency of the *k*-mer being extended: the higher the frequency, the higher the threshold. An adaptive threshold allows the assembly of both high and low coverage genomes, without the fragmentation that would result from a static value for $t_{hq}$.

Several additional steps are executed in an iteration to improve the quality of the contigs (steps 3 to 6 in Fig. 7). In step 3, short alternative sequences with the same start and end *k*-mers (*bubbles*) are merged and short dead-end forked sequences (*hairs*) are removed. Next, in iterative graph pruning (step 4), contigs on forks that differ from the depth of neighboring contigs are treated as errors and the connections are removed. Finally, in steps 5 and 6, the reads are aligned to the end of contigs and used to extend the contigs through localized assemblies, without the forks caused by global *k*-mer analysis^39^. All these refinements are parallelized by leveraging distributed graphs (implemented as distributed hash tables in UPC).

Finally, in step 7, the contigs produced in iteration *i* are used to generate a new set of longer $(k+s)$-mers (where *s* is the size of the increase in *k* in the next iteration). The contigs are treated as long error free reads and the longer $(k+s)$-mers are extracted from them, and added to the set used in the next iteration. Each of these added $(k+s)$-mers will have unique extensions on both sides.

### Scaffolding

The contigs that are produced by the iterative contig generation are joined together to form longer contigs (scaffolds) during a final scaffolding phase. The scaffolding algorithms described here are implemented in the *cgraph* module, which is a new development since the previous description of *MetaHipMer*^8^. The first step in scaffolding is to align the reads to the contigs, and use the resulting information to determine which contigs can connect together. The contigs and their connections form a *contig graph*, where the vertices are contigs and the edges are links between the contigs determined from the alignments. The edges can be derived both from single reads that overlap two contigs (a *splint*) or from a pair of reads, where one side of the pair aligns to one contig, and the other side to a second contig (a *span*). The scaffolding phase first builds the contig graph, and then traverses it to determine the paths that form scaffolds, which are contigs linked together with possible gaps between them. A final pass fills the gaps where possible using the reads that formed the edges.

The contig graph traversal follows an approach similar to that used by ExSPAnder^40^. When a traversal encounters a fork, it does a search of all possible paths out from that fork, up to a certain depth, looking for a unique contig (vertex) with a similar depth to the overall walk depth. If a unique contig is found, then the traversal will choose the appropriate edge out of the fork; otherwise the traversal terminates. Thus the traversal ends up connecting contigs of similar depth to form a longer path, and can span high-depth regions. There are several additional refinements that can further resolve forks when the depths are similar, for example, choosing much longer alignments, or choosing much better supported edges (where support is the number of reads that confirm an edge).

UPC++ is used to parallelize the graph traversal. First, two distributed hash tables are built, one storing the vertices and the other the edges. Once built, each process then iterates through its local portion of the vertices, starting a traversal from each one in turn, from the longest contig to the shortest (because longer contigs are more likely to have accurate depth and low error). Because the search for the next edge out of a fork is limited and depends on the depth of the starting vertex in the traversal, a vertex could end up being included in multiple different paths, started by different ranks. *MetaHipMer* resolves these conflicts with a speculative approach: each rank builds its own set of paths independently, and then discards paths than include a vertex that is included in another, longer path. This can result in vertices than are not included in any paths, so the process is repeated until no more starting vertices are available (they are all included in longer paths).

Finally, the whole process of aligning reads to contigs, building a contig graph, and traversing it to find longer contigs, can be repeated multiple times. Each repeat tends to increase the contiguity of the assembly, at a cost of an increase in errors. This presents a trade-off that the user can tune to achieve results that suit their goals. By default, the scaffolding is repeated twice.

## Data availability

The datasets supporting the conclusions of this article are described in Table 1 in the Supplementary information, with details about how to obtain every dataset.

*MetaHipMer* is available for public use under an open source license, and can be downloaded from https://sourceforge.net/projects/hipmer.

## Supplementary information

Supplementary information.
