# Supplementary material for: Terabase-scale metagenome coassembly with MetaHipMer
Source: Sci Rep. 2020 Jul 1;10:10689. doi: 10.1038/s41598-020-67416-5 (PMC7329831; doi:10.1038/s41598-020-67416-5)
Supplement: Supplementary file 1 — Supplementary information. [file 41598_2020_67416_MOESM1_ESM.pdf]

Supplementary Information
Terabase-scale metagenome coassembly with MetaHipMer

Steven Hofmeyr[1,*], Rob Egan[2], Evangelos Georganas[3], Alex C Copeland[2], Robert Riley[2], Alicia Clum[2], Emiley Eloe-Fadrosh[2], Simon Roux[2], Eugene Goltsman[2], Aydın Buluç[1,4], Daniel Rokhsar[2,5], Leonid Oliker[1], and Katherine Yelick[1,4]

[1]Computational Research Division, Lawrence Berkeley National Laboratory, Berkeley CA 94720, USA
[2]Joint Genome Institute, Lawrence Berkeley National Laboratory, Berkeley CA 94720, USA
[3]Parallel Computing Lab, Intel Corp., Santa Clara CA 95054, USA
[4]Department of Electrical Engineering and Computer Sciences, University of California, Berkeley CA 94720, USA
[5]Department of Molecular and Cellular Biology, University of California, Berkeley CA 94720, USA
[*]shofmeyr@lbl.gov

ABSTRACT

Metagenome sequence datasets can contain terabytes of reads, too many to be *coassembled* together on a single shared-memory computer; consequently, they have only been assembled sample by sample (*multiassembly*) and combining the results is challenging. We can now perform coassembly of the largest datasets using *MetaHipMer*, a metagenome assembler designed to run on supercomputers and large clusters of compute nodes. We have reported on the implementation of *MetaHipMer* previously; in this paper we focus on analyzing the advantages of very large coassembly. In particular, we show that coassembly recovers a larger genome fraction than multiassembly and enables the discovery of more complete genomes, with lower error rates. We present several assemblies of terabyte datasets that have never been coassembled before, demonstrating the benefit of the *MetaHipMer* coassembly approach. *MetaHipMer* is available for public use under an open source license and all datasets used in the paper are available for public download.

1 Command lines for software used

Note: all words in capitals starting with a $ are user-defined variables, e.g. `$READ_LENGTH` is a read length, such as 150.

1.1 Mason

Version: 2.0.9
Available from: https://github.com/seqan/seqan/tree/master/apps/mason2
Command line:

```
mason_simulator --illumina-read-length $READ_LENGTH -ir $REFERENCE_FILE \
  -n $NUMBER_PAIRS --fragment-mean-size $INSERT_SIZE_MEAN \
  --fragment-size-std-dev $INSERT_SIZE_STDDEV -o $OUT_1.fq -or $OUT_2.fq
```

Where the number of pairs is determined from the desired depth by:

```
NUMBER_PAIRS = $REFERENCE_LENGTH * $CHOSEN_DEPTH / $READ_LENGTH
```

1.2 CAMISIM

Version: 0.0.6
Available from: https://github.com/CAMI-challenge/CAMISIM
Command line:

```
metagenomesimulation.py arctic_synth_config.ini
```

Where the configuration file `arctic_synth_config.ini` is:

```
[Main]
seed=632741178
phase=0
```

```
max_processors=1
dataset_id=RL
output_directory=arctic_synth/arctic_samples_replicates
temp_directory=/dev/shm
gsa=False
pooled_gsa=False
anonymous=False
compress=0

[ReadSimulator]
readsim=tools/art_illumina-2.3.6/art_illumina
error_profiles=tools/art_illumina-2.3.6/profiles
samtools=tools/samtools-1.3/samtools
profile=mbarc
size=0.4
type=art
fragments_size_mean=270
fragment_size_standard_deviation=30

[CommunityDesign]
distribution_file_paths=
ncbi_taxdump=mar_ref/ncbi_taxonomy
strain_simulation_template=scripts/StrainSimulationWrapper/sgEvolver/simulation_dir
number_of_samples=12

[community0]
metadata=arctic_synth/arctic-species-metadata.tsv
id_to_genome_file=arctic_synth/arctic-species.tsv
id_to_gff_file=
genomes_total=25
genomes_real=25
max_strains_per_otu=1
ratio=1
mode=replicates
log_mu=1
log_sigma=1
gauss_mu=1
gauss_sigma=1
view=False
```

1.3 Quast

Version: 5.0.0
Available from: http://quast.sourceforge.net/quast.html
Command line:

```
metaquast.py --rna-finding --no-icarus --fragmented -t 80 -o $OUTPUT \
  -r $REFERENCE_FASTQ $ASSEMBLED_FASTA
```

The number of complete rRNAs (16S and 23S) are counted with the following bash commands:

```
grep "product=16S" $GFF_FILE |grep -v partial|wc -l
grep "product=23S" $GFF_FILE |grep -v partial|wc -l
```

where `$GFF_FILE` is found in the metaquast output at

```
combined_reference/predicted_genes/$ASSEMBLER_NAME.rna.gff
```

1.4 MetaBAT2

Version: 2.13-29-g2e72973
Available from: https://bitbucket.org/berkeleylab/metabat
Command line:

```
metabat2 -i $READS -o $OUTDIR/bin
```

1.5 CheckM

Version: 1.0.13
Available from: https://ecogenomics.github.io/CheckM
Command line:

```
checkm lineage_wf -f $OUTDIR/CheckM.txt -t $NUM_CORES -x fa $OUTDIR $OUTDIR/SCG
```

where `OUTDIR` is the MetaBAT2 output directory.
The results were analyzed for completeness and contamination using the following R script:

```
#!/usr/bin/env Rscript
args = commandArgs(trailingOnly=TRUE)
source('http://portal.nersc.gov/dna/RD/Metagenome_RD/MetaBAT/Files/benchmark.R')
printPerf(list(calcPerfBySCG(paste(args[1], "/CheckM.txt", sep=''), removeStrain=T)),
          rec=c(seq(.1,.9,.1),.95), prec=c(seq(.6,.9,.1),.95,.99))
```

2 Assemblers

2.1 MetaHipMer

Version: 1.2.1
Available from: https://sourceforge.net/projects/hipmer
Command line:

```
hipmer --meta -i $READS
```

2.2 MEGAHIT

Version: 1.1.3
Available from: https://github.com/voutcn/megahit
Command line:

```
megahit --12 $READS
```

2.3 metaSPAdes

Version: 3.13.0
Available from: http://cab.spbu.ru/software/meta-spades/
Command line:

```
metaspades.py --pe1-12 $READS --only-assembler -t $NUM_CORES
```

3 Datasets

See Tables S1 and S2 for details on all the datasets. Further details for *ArcticSynth* can be found in Tables S3 and S4.

Dataset	Description	Author/PI	Reference
Wetlands	Wetland surface sediment. Metagenomics sequences from a time-series of wetlands soil samples collected from several physical sites in the Twitchell Wetlands in the San Francisco Bay-Delta.	S. Tringe	S. Theroux, et al. "Microbial diversity in restored wetlands of San Francisco Bay." American Geophysical Union, Fall Meeting 2013, abstract id. B33A-0463 (2013).
Cow Rumen	A collection of metagenomic DNA sequenced from microbes adherent to plant fiber incubated in the cow rumen.	M. Hess	M. Hess, et al. "Metagenomic discovery of biomass-degrading genes and genomes from cow rumen." Science 331(6016), 463–467 (2011)
Soil Carbon	Metagenome DNA sequenced for a project that aims to identify and characterize the dominant uncultivated microorganisms that mediate major transformations in the soil carbon cycle.	D. Buckley	Unpublished; JGI CSP 503502: Microbial metabolic dependency and its impacts on the soil carbon cycle
WA	Marine microbial communities from the Western Arctic Ocean.		
Marine	Tara Oceans polar circle sample.		
Gut	Human gut metagenome sample.		
MBARC-26	Synthetic high-depth, simple dataset composed of 23 bacterial and 3 archaeal strains with finished genomes that span 10 phyla and 14 classes, a range of GC contents, genome sizes, repeat content, and that encompass a diverse abundance profile.	E. Singer	E. Singer, et al. "Next Generation Sequencing Data of a Defined Microbial Mock Community." Scientific Data 3 (September). Nature Publishing Group: 160081. (2016) doi:10.1038/sdata.2016.81.
ArcticSynth	Synthetic metagenome sample of 25 genomes matching Western Arctic Ocean (see Tables S3 and S4	S. Hofmeyr	

Table S1. Description of datasets.

Dataset	Platform	Size (GB)	# Reads	Gbp	SRA	URL
Wetlands	Illumina GAII	2637	7467203592	1120	SRR1182407 SRR1184661 SRR403474 SRR404111 SRR404117 SRR404119 SRR404151 SRR404204 SRR407529 SRR407548 SRR407549 SRR410821 SRR437909 SRR5198900 SRR5198901 SRR5198902 SRR5198903 SRR5246785 SRR5246787 SRR5246790 SRR5246791 SRR6203186	
Cow Rumen	Illimina HiSeq	2663	7922832126	1188		https://portal.nersc.gov/dna/RD/CowRumen/CowRumenRaw/
Soil Carbon	Illimuna NovaSeq	3343	9901426776	1471		
WA	Illimuna HiSeq 2500	822	2465328090	370	SRR5819383 SRR5819595 SRR5819381	https://gold.jgi.doe.gov/study?id=Gs0118432
Marine	Illimuna HiSeq 2500	100.6	478583618	48	ERR2762185	https://portal.nersc.gov/project/hipmer/MetaHipMer_datasets_12_2019/
Gut	Illimuna HiSeq 2000	7.9	30169866	3	SRR769529	https://portal.nersc.gov/project/hipmer/MetaHipMer_datasets_12_2019/
MBARC-26	HiSeq-HO 2000	31.9	173981994	52	SRX1836716	
ArcticSynth	Simulated	9.9	31999090	2.3	See Table S4	https://portal.nersc.gov/project/hipmer/MetaHipMer_datasets_12_2019/

Table S2. Details of datasets.

**Genome**	**Abundances per Sample**												Depth
Arcticibacterium luteifluviistationis	0.0031	0.0206	0.0062	0.0003	0.0019	0.0102	0.0042	0.0177	0.0189	0.0121	7e-06	0.0109	10.4
Candidatus Nitrosopumilus sp AR2	0.0033	0.0245	0.0185	0.0128	0.0059	0.0055	7e-06	0.0023	0.0029	0.0161	0.0155	0.0165	12.2
Colwellia psychrerythraea 34H	0.0907	0.0821	0.0798	0.0846	0.0868	0.0724	0.0803	0.0729	0.0763	0.0857	0.0663	0.0808	93.8
Colwellia sp Arc7-D MMP08912473	0.0060	0.0091	0.0160	0.0133	0.0115	0.0158	0.0029	0.0115	0.0232	0.0144	0.0111	0.0010	13.3
Denitrovibrio acetiphilus DSM 12809	0.0108	0.0135	0.0144	0.0092	0.0128	0.0226	0.0165	0.0109	0.0227	0.0140	0.0224	0.0285	19.4
Desulfotalea psychrophila LSv54	0.0942	0.0785	0.0927	0.0865	0.0807	0.0860	0.0872	0.0785	0.0871	0.0926	0.0851	0.0877	101.5
Flavobacterium arcticum	0.0369	0.0367	0.0439	0.0495	0.0479	0.0445	0.0398	0.0376	0.0241	0.0325	0.0539	0.0348	47.3
Kosmotoga olearia TBF 1951	0.0077	0.0138	0.0098	0.0159	0.0109	0.0143	0.0085	0.0205	0.0109	0.0117	0.0079	0.0198	14.9
Marinobacter psychrophilus 20041	0.0028	0.0087	7e-06	0.0078	0.0147	0.0120	0.0019	0.0070	0.0115	0.0147	0.0053	0.0067	9.2
Marinobacter sp Arc7 DN 1	0.0495	0.0469	0.0528	0.0628	0.0493	0.0490	0.0447	0.0595	0.0459	0.0521	0.0459	0.0589	60.5
Marinobacter sp BSs20148	0.0185	0.0199	0.0278	0.0183	0.0221	0.0182	0.0172	0.0264	0.0352	0.0143	0.0207	0.0231	25.7
Microbacterium sp LKL04	0.1485	0.1298	0.1305	0.1265	0.1511	0.1340	0.1276	0.1311	0.1308	0.1251	0.1186	0.1169	153.7
Nocardiopsis dassonvillei NOCA502F	0.0143	0.0200	0.0017	0.0236	0.0135	0.0132	0.0239	0.0291	0.0061	0.0152	0.0165	0.0178	19.1
Octadecabacter arcticus 238	0.0140	0.0274	0.0151	0.0087	0.0214	0.0165	0.0260	0.0218	0.0080	0.0053	0.0228	0.0297	21.2
Paraglaciecola psychrophila 170	0.0663	0.0503	0.0598	0.0667	0.0577	0.0691	0.0588	0.0637	0.0572	0.0601	0.0657	0.0555	71.6
Polaribacter sp ALD11	0.0091	0.0101	0.0188	0.0190	0.0063	0.0187	0.0190	0.0194	0.0148	0.0179	0.0260	0.0084	18.3
Pseudoalteromonas aliena EH1	0.0200	0.0190	0.0153	0.0117	0.0307	0.0166	0.0327	0.0317	0.0214	0.0283	0.0323	0.0312	28.4
Psychrobacter sp P11G3	0.0134	0.0179	0.0162	0.0156	0.0166	0.0156	0.0210	0.0087	0.0191	0.0106	0.0186	0.0053	17.5
Psychromonas ingrahamii 37	0.0743	0.0698	0.0603	0.0657	0.0700	0.0657	0.0755	0.0761	0.0744	0.0708	0.0732	0.0682	82.6
Rhodobacteraceae bacterium BAR1	0.0488	0.0421	0.0530	0.0401	0.0439	0.0519	0.0425	0.0501	0.0534	0.0475	0.0520	0.0549	56.8
Rhodococcus sp B7740	0.0317	0.0357	0.0279	0.0354	0.0241	0.0274	0.0364	0.0310	0.0319	0.0340	0.0302	0.0315	36.9
Salegentibacter sp T436	0.0190	0.0163	0.0308	0.0146	0.0245	0.0135	0.0185	0.0222	0.0171	0.0228	0.0115	0.0271	23.3
Sulfitobacter sp SK011	0.1257	0.1190	0.1173	0.1149	0.1070	0.1094	0.1059	0.1016	0.1152	0.1090	0.1133	0.0915	130.1
Thalassolituus oleivorans R6-15	0.0783	0.0656	0.0614	0.0741	0.0744	0.0808	0.0802	0.0595	0.0792	0.0780	0.0626	0.0784	85.4
Thalassolituus oleivorans strain K188	0.0127	0.0220	0.0295	0.0221	0.0138	0.0166	0.0284	0.0085	0.0121	0.0148	0.0222	0.0143	21.2

Table S3. Abundances and depths of the 12 replicate samples for *ArcticSynth*.

**Genome**	**Refseq identifiers**
Arcticibacterium luteifluviistationis	NZ_CP029480.1
Candidatus Nitrosopumilus sp AR2	CP003843.1
Colwellia psychrerythraea 34H	CP000083.1
Colwellia sp Arc7-D MMP08912473	CP028924.1
Denitrovibrio acetiphilus DSM 12809	CP001968.1
Desulfotalea psychrophila LSv54	CR522870.1 CR522871.1 CR522872.1
Flavobacterium arcticum	NZ_CP031188
Kosmotoga olearia TBF 1951	CP001634.1
Marinobacter psychrophilus 20041	NZ_CP011494.1
Marinobacter sp Arc7 DN 1	NZ_CP031848.1
Marinobacter sp BSs20148	CP003735.1
Microbacterium sp LKL04	LT627736.1
Nocardiopsis dassonvillei NOCA502F	NZ_CP017965.1
Octadecabacter arcticus 238	CP003742.1 CP003743.1 CP003744.1
Paraglaciecola psychrophila 170	NC_020514.1
Polaribacter sp ALD11	CP025119.1
Pseudoalteromonas aliena EH1	NZ_CP019628.1
Psychrobacter sp P11G3	NZ_CM003596.1 NZ_CM003597.1 NZ_CM003598.1 NZ_CM003599.1 NZ_CM003600.1
Psychromonas ingrahamii 37	CP000510.1
Rhodobacteraceae bacterium BAR1	NZ_CP032125.1
Rhodococcus sp B7740	NZ_CP010797.1
Salegentibacter sp T436	NZ_CP012872.1
Sulfitobacter sp SK011	NZ_CP025803.1
Thalassolituus oleivorans R6-15	CP006829.1
Thalassolituus oleivorans strain K188	NZ_CP017810.1

Table S4. NCBI Refseq accession identifiers for ArcticSynth.
